# Ultrasonic-Assisted Surface Finishing of STAVAX Mold Steel Using Lab-Made Polishing Balls on a 5-Axis CNC Machining Center

**DOI:** 10.3390/ma16175888

**Published:** 2023-08-28

**Authors:** Fang-Jung Shiou, Jian-Nan Pan, Zhao-Li Ding, Sun-Peng Lin

**Affiliations:** Department of Mechanical Engineering, National Taiwan University of Science and Technology, Taipei 106, Taiwan; m10503232@mail.ntust.edu.tw (J.-N.P.); m10303247@mail.ntust.edu.tw (Z.-L.D.); splin@mail.ntust.edu.tw (S.-P.L.)

**Keywords:** ultrasonic vibration-assisted polishing, lab-made polishing ball, multiple pass polishing, 5-axis CNC machining center, volumetric wear

## Abstract

The inconvenience of conventional wool ball polishing is that the surface finishing process should be equipped with a slurry container. The main objective of this research is to develop an ultrasonic-assisted surface finishing process for STAVAX mold steel on a 5-axis CNC machining center, by using new lab-made rubber polishing balls containing the abrasive aluminum oxide instead of the traditional wool ball polishing. In total, five types (type A to type E) of new rubber-matrixed polishing balls with a composite of nitrile butadiene rubber (NBR), an abrasive of aluminum oxide, and an additive of silicon dioxide have been developed. The performance of the composites with different grain sizes (0.05 μm to 3 μm) and concentrations of the abrasive of aluminum oxide have been investigated. The effects of multiple polishing passes on the surface roughness improvement for the lab-made polishing balls have also been investigated in this study. A surface roughness of Ra 0.027 μm on average was achieved by using the multiple polishing process of E-C-B-A. The volumetric wear of the lab-made polishing balls, using ultrasonic vibration-assisted polishing, can be improved from about 12.64% (type A) to 65.48% (type E) compared with the non-vibration-assisted polishing. The suitable combination of the ultrasonic vibration-assisted polishing parameters were an amplitude of 10 μm, a frequency of 23 kHz, a spindle speed of 5000 rpm, a feed rate of 60 mm/min, a stepover of 20 μm, a penetration depth of 180 μm, and a polishing pass of E-C-B-A, based on the experimental results. The surface roughness improvement on a test carrier with a saddle surface has also been presented by using the ultrasonic vibration-assisted polishing with the lab-made polishing balls.

## 1. Introduction

Ultrasonic waves have been used for numerous purposes in many different applications, ranging from the non-destructive inspection of materials to the ultrasonic-assisted chemical synthesis of materials and welding in recent years [1]. Ultrasonic-assisted welding is one of the important applications of ultrasonic technology with a high frequency (15 kHz to 40 kHz) and a low amplitude [2]. Different kinds of ultrasonic vibration-assisted machining processes, such as milling [3,4], turning [5,6], grinding [7,8], drilling [9,10], deburring [11,12], diamond machining (cutting) [13,14], electrical discharge machining (EDM) [15,16], abrasive water jet machining [17], hybrid laser- and ultrasonic-assisted machining [18,19], and surface finishing [20,21,22], etc., have been developed in recent years. The materials processed by the ultrasonic-assisted machining processes [3,4,5,6,7,8,9,10,11,12,13,14,15,16,17,18,19,20,21,22], in general, can be classified as brittle materials, hard-machining metallic materials, composite materials, and non-metallic materials, etc.

The investigation into cutting performance in the ultrasonic-assisted helical milling of Ti6Al4V alloy by various parameters and cooling strategies has been presented in [3]. The study of the mechanism of burr formation by simulation and experiments on ultrasonic vibration-assisted micro-milling has been presented in [4]. The benefits of green ceramic machining through ultrasonic-assisted turning have been experimentally investigated in [5]. Sustainable cooling strategies to reduce tool wear, power consumption, and surface roughness during the ultrasonic-assisted turning of Ti-6Al-4V have been proposed in [6]. The damage characteristics of ultrasonic vibration-assisted grinding of a C/SIC composite material has been studied in [7]. The effects of processing parameters on the surface quality of wrought Ni-based superalloy by ultrasonic-assisted electrochemical grinding has been investigated in [8]. The chip generation mechanism of Inconel 718 with ultrasonic-assisted drilling by step drill has been studied in [9]. The experimental research on new hole-making methods assisted with asynchronous mixed-frequency vibration in TiBw/TC4 composites has been reported in [10]. The ultrasonic-assisted abrasive micro-deburring of micromachined metallic alloys has been studied in [11]. The burr removal from high-aspect-ratio micro-pillars using ultrasonic-assisted abrasive micro-deburring has been discussed in [12]. The enhanced machinability of Ni-based single crystal superalloy by vibration-assisted diamond cutting has been presented in [13]. The zirconia responses to edge chipping damage induced in conventional and ultrasonic vibration-assisted diamond machining are presented in [14]. Wire-EDM performance has been enhanced with zinc-coated brass wire electrode and ultrasonic vibration [15]. The research on ultrasonic vibration-assisted electrical discharge machining SiCp/Al composite has been presented in [16]. A numerical modeling and experimental study on the material removal process using ultrasonic vibration-assisted abrasive water jet has been reported in [17]. The hybrid simultaneous laser- and ultrasonic-assisted machining of the Ti-6Al-4V alloy has been presented in [18]. The comparative study of the laser and ultrasonic vibration-assisted hybrid turning of micro-SiCp/AA2124 composites has been carried out in [19].

To improve the surface roughness of the test objects, some vibration-assisted surface polishing devices have been developed in [20,21,22]. The experimental study on ultrasonic-assisted electrolyte plasma polishing of SUS304 stainless steel has been presented in [20]. The modeling and analysis of the material removal rate for the ultrasonic vibration-assisted polishing of optical glass BK7 has been proposed in [21]. The ultrasonic-assisted innovative polyurethane tool to polish the mold steel has been developed in [22]. A vibration-assisted ball polishing device was developed in [23] to obtain the ultra-precision surface finish of the hardened stainless mold steel. The ball burnishing process has also been introduced to perform the pre-finishing process for ball polishing to improve the surface roughness of the stainless mold steel in [23]. The wear reduction of the machining tool can be achieved by using ultrasonic-assisted devices (tools) [6,23,24,25]. To evaluate the surface quality of a product, some definitions and surface texture parameters, such as the height, spatial, and hybrid parameters, etc., can be found in ISO 21920-2:2021 [26]. The functional importance of the surface texture parameters, including both the amplitude parameters of the surface and the functional parameters, has been presented in [27].

The effect of aluminum powder on the properties of nitrile rubber (NBR) composites and the role of the bonding agent, viz., hexamethylene tetramine-resorcinol, has been investigated in [28]. The polymethyl methacrylate (PMMA) denture base composites were enhanced by various combinations of nitrile butadiene rubber (NBR) and treated ceramic fillers (aluminum oxide, yttria-stabilized zirconia, and silicon dioxide) reported in [29].

**Figure 1:**
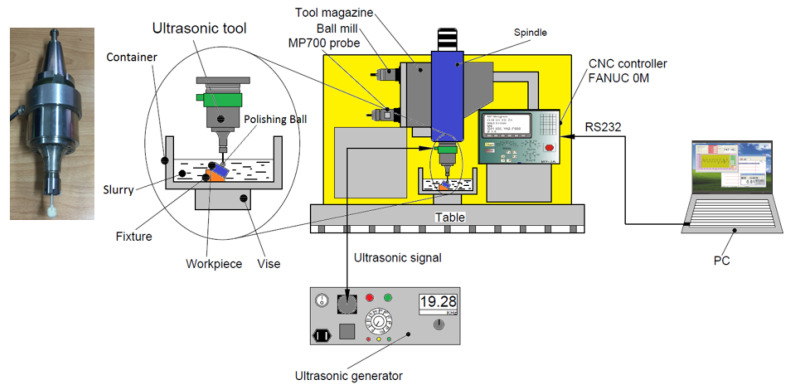
Experimental setup of the ultrasonic-assisted polishing tool integrated in a CNC machining center.

Vibration-assisted ball polishing using the ultrasonic tool with the holder type BT40, which can be integrated into the tool magazine of a machining center, has been implemented in [30], as shown in Figure 1. The processing parameters for the STAVAX mold steel, such as the amplitude, frequency, spindle speed, abrasive diameter, feed rate, depth of penetration, etc., have been investigated in [30]. To facilitate the ultrasonic-assisted ball polishing process without using a slurry container device and concerning green manufacturing, the new polishing balls embedded with the abrasive materials are to be introduced in this study.

The main objective of this study was to present the development of the new lab-made polishing balls, a different number of passes of polishing balls on the surface roughness improvement, and to investigate the tool wear of the polishing ball using the ultrasonic vibration-assisted ball polishing on a machining center. The property of the tested material, STAVAX stainless mold steel, which is equivalent to AISI 420 modified [31], the experimental setup of the ultrasonic-vibration-assisted ball polishing system on a 5-axis machining center, and the investigation of the used ultrasonic tool are introduced in Section 2. The development of the new lab-made polishing balls, the suitable combination of the ultrasonic vibration-assisted ball polishing parameters for a plane surface, the effects of different passes on the surface roughness improvement, the volumetric wear of the polishing balls, and the application to the surface finishing of a test carrier with a saddle surface are presented in Section 3. Some discussion and future work will be explained in Section 4. Finally, some remarkable results of this work are concluded.

## 2. Materials and Methods

### 2.1. Materials

STAVAX is a premium-grade stainless tool steel (AISI 420 modified) with the following properties [31]:

- Good corrosion resistance
- Excellent polishability
- Good wear resistance
- Good machinability
- Good stability in hardening

The combination of these properties yields a steel with outstanding production performance. As a result, it is suitable for application to most molds of larger tools. The chemical composition of the STAVAX stainless mold steel is shown in Table 1 [31]. The hardness of the STAVAX used in this study is about HRB190 (HRC 22) after soft annealing.

### 2.2. Experimental Setups on a 5-Axis Machining Center

**Figure 2:**
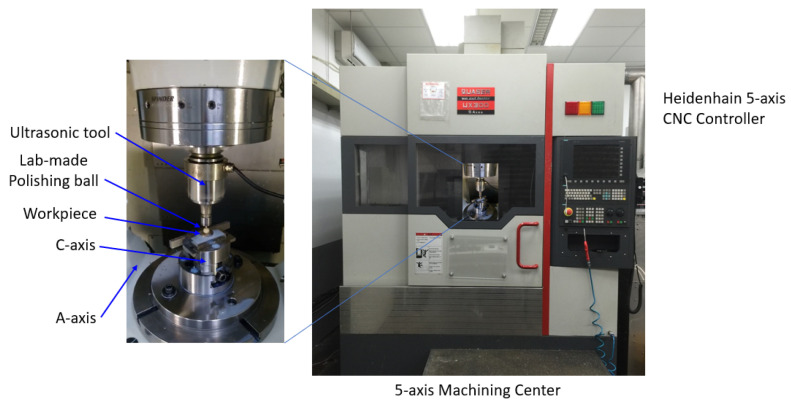
Photo of the ultrasonic vibration-assisted ball-polishing tool mounted on the 5-axis machining center.

To investigate the performance of the ultrasonic vibration-assisted polishing using lab-made polishing balls, the experimental setup has been developed, as shown in Figure 2. The developed lab-made polishing ball is clamped on the ultrasonic tool with the holder type BT40 that can be mounted on the spindle of the machining center. The 5-axis machining center used in this research was a product of the QUASER Co., (Taichung, Taiwan) type UX300. The machining center equipped with the CNC controller of the HEIDENHAIN Company (Traunreut, Germany), type iTNC 530. A TS740 touch-trigger probe, product of HEIDENHAIN Company (Traunreut, Germany), was integrated with the machining center tool magazine to measure the origin coordinates of the workpiece to be fabricated and to execute the in-process measurement. To simulate the sequence of fine milling, ball burnishing, and ball polishing path and to generate the respective NC codes, the Unigraphics NX10 CAD/CAM software (NX10) has been used. The Hommelwerke T8000 roughness instrument, a product of JENOPTIC Co. (Jena, Germany), was used to measure the surface roughness of the machined specimens.

### 2.3. Specification and Investigation of the Used Ultrasonic Tool

An ultrasonic tool, fabricated by KLI Technology [32], was adopted in this study. The detailed specification of the ultrasonic tool used is listed in Table 2.

**Figure 3:**
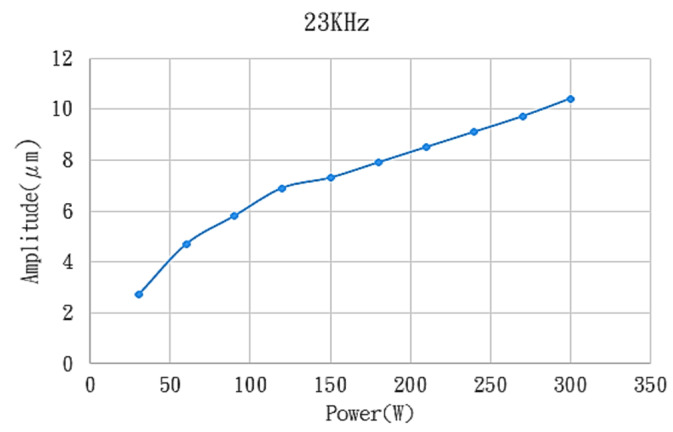
Measured results of the vibration amplitudes of the ultrasonic tool under different power.

The LK-H025 triangulation laser probe, a product of KEYENCE Corporation (Osaka, Japan), has been used to measure the amplitude of the ultrasonic tool. According to the test results, the greater the working frequency, the greater the generated amplitudes of the ultrasonic tool by fixing the driven power of the controller. As a result, the suitable working frequency of 23 kHz has been set. The measured amplitudes of the ultrasonic tool with the power ranging from 60 W to 300 W and with the fixed working frequency of 23 kHz is shown in Figure 3. The relationship between the amplitude and the power was almost linear.

## 3. Results

### 3.1. Development of the Lab-Made Polishing Balls

#### 3.1.1. Design and Fabrication of the Mold for the Lab-Made Polishing Balls

**Figure 4:**
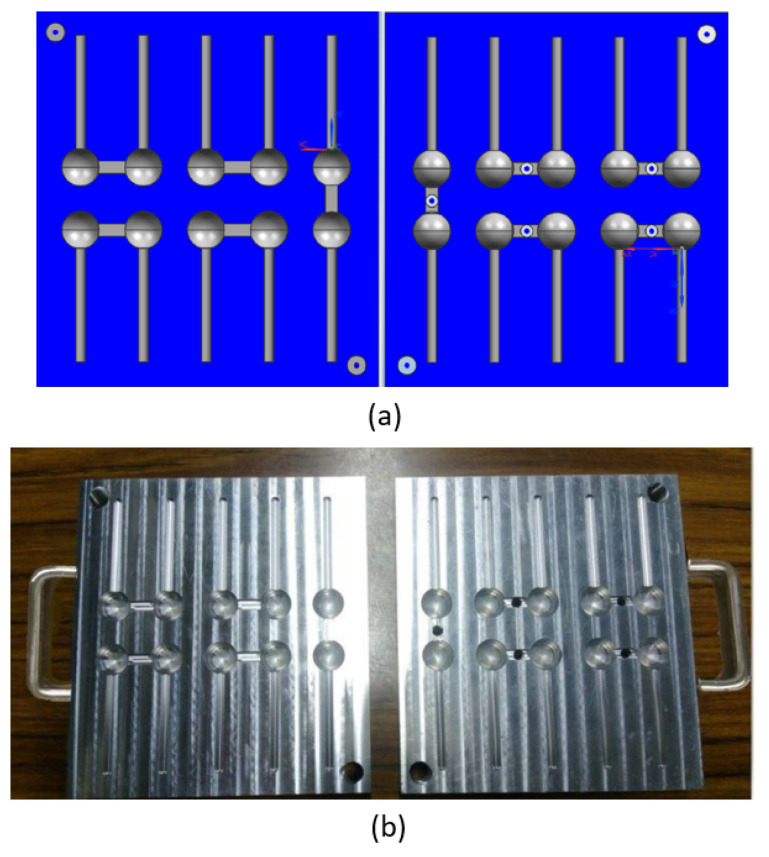
(**a**) Design of the polishing balls using NX 10; (**b**) fabricated mold for thermal pressing.

A mold made of Al-6061T6 with the hardness of HRB 54 has been designed and fabricated to generate the polishing balls with a diameter of 12 mm, as shown in Figure 4.

#### 3.1.2. Configuration and Fabrication of the Lab-Made Polishing Balls

Considering the slurry container that is no longer being used, new polishing balls embedded with abrasives were developed in this study. The abrasive aluminum oxide (Al_2_O_3_) is suitable for polishing the stainless mold steel, according to the previous study results [23,30]. As a result, the new polishing balls embedded with aluminum oxide abrasive has been developed by taking the Nitrile Butadiene Rubber (NBR) as the matrix, mixing it with the aluminum oxide abrasive with different grain sizes (0.05 μm to 3 μm) and concentration and additives of silicon dioxide. The powder components for the new polishing balls were Nitrile Butadiene Rubber (NBR) (60, 60, 50, 60, and 50 wt%), aluminum oxide abrasives of various sizes (0.05 um to 3 m) (20, 20, 30, 20, and 40 wt%), and silicon dioxide (20 wt%). The concentration and abrasive size of aluminum oxide is shown in Table 3. Concerning the good physical, mechanical, and chemical properties, such as abrasion resistance, adhesion to metal, compression set, tear resistance, vibration dampening, and solvent resistance, etc., NBR was selected as the matrix material. Five types of NBR-based blanks for the polishing balls were made after the NBR, abrasives, and some additives were homogenized by a blending machine. The hardness of each blank has been tested by using the Shore hardness tester. After the tensile test specimens were made from the NBR-based blanks, the yielding strength and the static coefficient of friction have been tested by using a universal material testing machine. The measured results of the hardness, yielding strength, and the static coefficient of friction have been summarized in Table 4.

**Figure 5:**
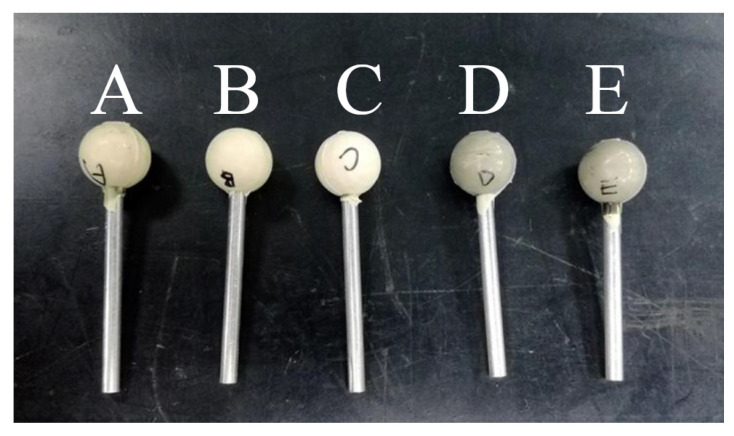
Fabricated polishing balls.

Different kinds of polishing balls with the diameter of 12 mm have been fabricated by the thermal forming processes, as shown in Figure 5.

### 3.2. Processing Parameters for the Ultrasonic Tool

To perform the surface finishing of the specimens and test carrier, the sequential ball burnishing and ultrasonic vibration-assisted polishing processes using the lab-made polishing balls have been adopted in this research. The adopted suitable burnishing parameters were the burnishing force of 80N, the feed rate of 300 mm/min, and the stepover of 30 μm. The burnished surface roughness of the fine-milled specimens was 0.11 μm using the suitable combination of the ball burnishing parameters. The suitable processing parameters using the ultrasonic tool for the STAVAX mold steel, such as the amplitude, frequency, spindle speed, abrasive diameter, feed rate, depth of penetration, etc., have been determined by Taguchi’s experimental method in [30]. An L18 (2^1^ × 3^7^) orthogonal table with one factor for two levels and seven factors for three levels has been figured out, to obtain the suitable processing parameters for the ultrasonic vibration-assisted ball polishing of STAVAX stainless steel. The configuration table is shown in Table 5. The amplitude, frequency, and spindle speed for the ultrasonic tool, abrasive diameter, feed rate, stepover, depth of concentration, and abrasive concentration have been selected as main factors and studied.

According to the S/N ratio calculation of the Taguchi L18 matrix experimental results, a suitable combination of the ultrasonic vibration-assisted ball polishing parameters is summarized in Table 6. The amplitude of the ultrasonic tool was 10 μm; the working frequency of the ultrasonic tool was 23 kHz; the spindle speed was 5000 rpm; the diameter of the aluminum oxide (Al_2_O_3_) was 0.3 μm; the feed rate was 60 mm/min; the stepover distance was 20 μm; the depth of penetration was 180 μm; and the slurry concentration was 1:10. Some of the parameters are to be applied to the experiments to the number of the passes of the lab-made polishing balls on the surface roughness improvement.

### 3.3. Effects of Different Passes on the Surface Roughness Improvement

**Figure 6:**
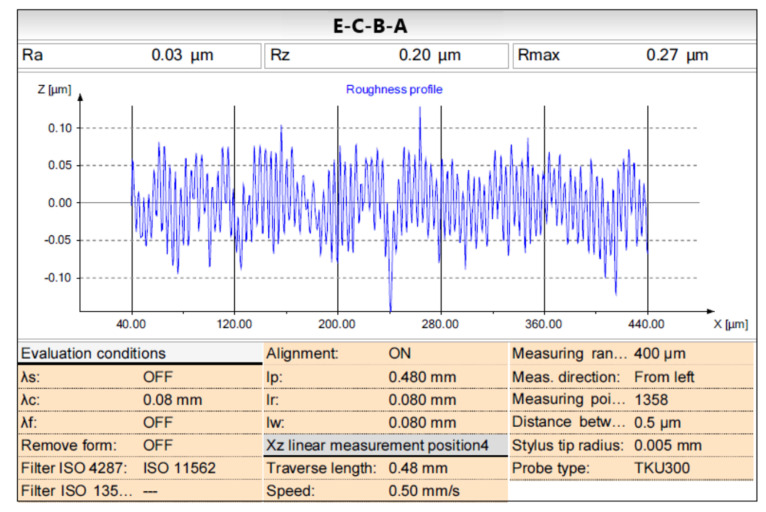
Measured surface roughness of the specimen using the combined E-C-B-A ultrasonic-assisted process parameters for the lab-made polishing balls.

Multiple polishing passes are needed to improve the surface roughness by reducing the grain size of the abrasives step by step, in general, to achieve the final surface finish for a precision mold with freeform surface in industrial application. The effects of different passes on the surface roughness improvement for the lab-made polishing balls has been investigated in this study. Two individual pass and four kinds of combined passes, namely Type E, Type D, Type E-A, Type E-C, Type E-C-B, and Type E-C-B-A, have been figured out and tested, as shown in Table 7, to sequentially reduce the grain size of the abrasives. According to the experimental results, the fine-milled and burnished surface with a surface roughness of Ra 0.13 μm could be improved to 0.027 μm on average using the combined type E-C-B-A passes. The measured surface roughness Ra 0.03 μm (Rz 0.20 μm) of the specimen using the combined E-C-B-A ultrasonic-assisted process parameters for the lab-made polishing balls is shown in Figure 6. As a result, to reduce the polishing time and obtain a good surface roughness of Ra 0.027 μm, the combination of the ultrasonic-assisted process parameters for the lab-made polishing balls is listed in Table 8. To reduce the polishing time, the surface roughness of Ra 0.04 μm on average was achievable by utilizing the Type E-C-B pass.

### 3.4. Volumetric Wear Improvement of the Lab-Made Polishing Balls

**Figure 7:**
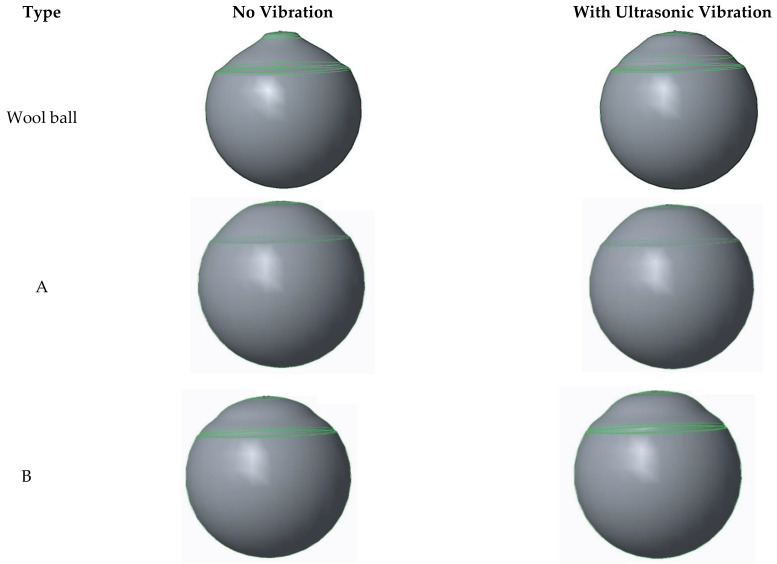

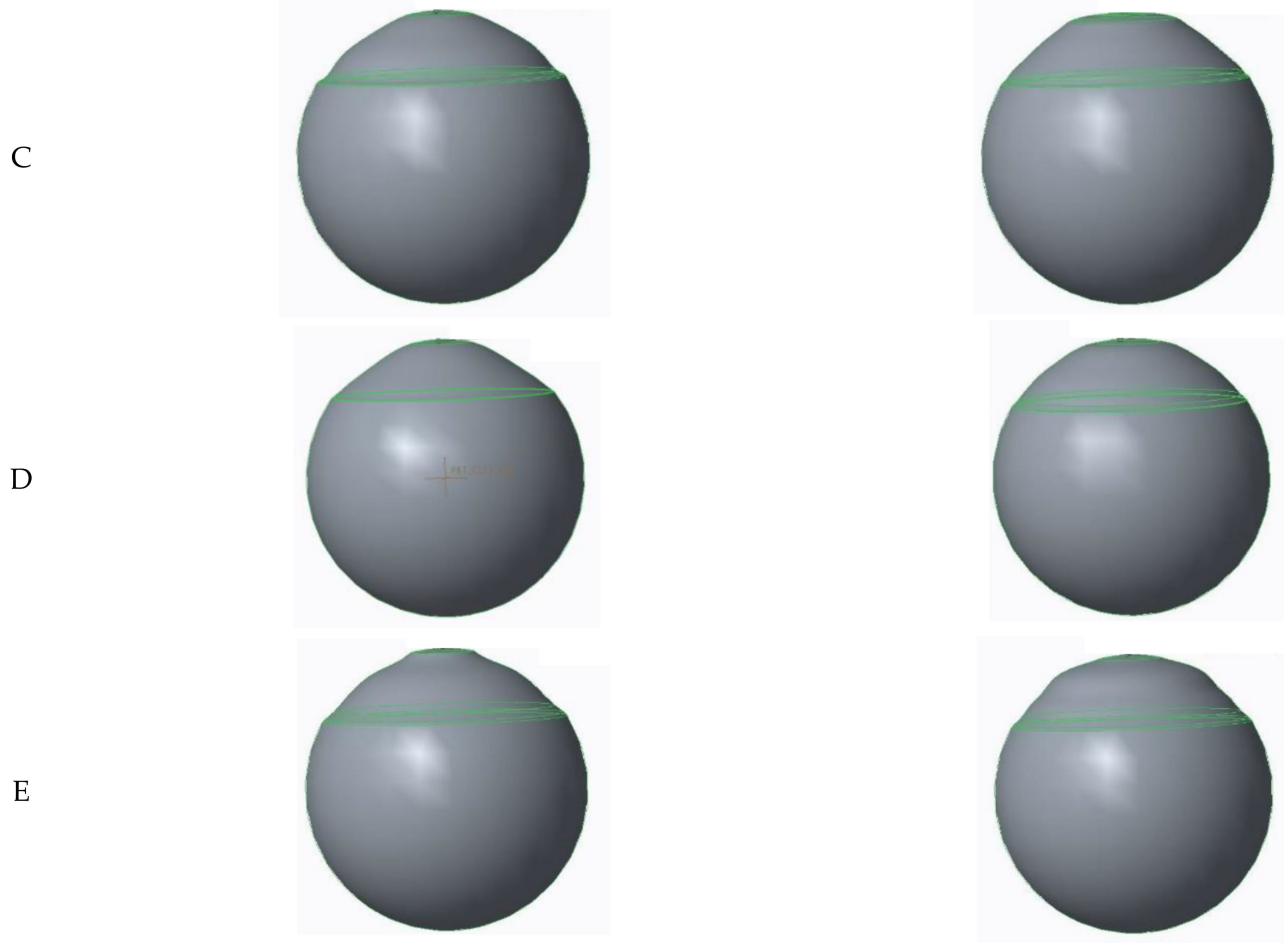
Constructed CAD models of the volumetric wear of different polishing balls from Type A to E explained in Table 9.

**Figure 8:**
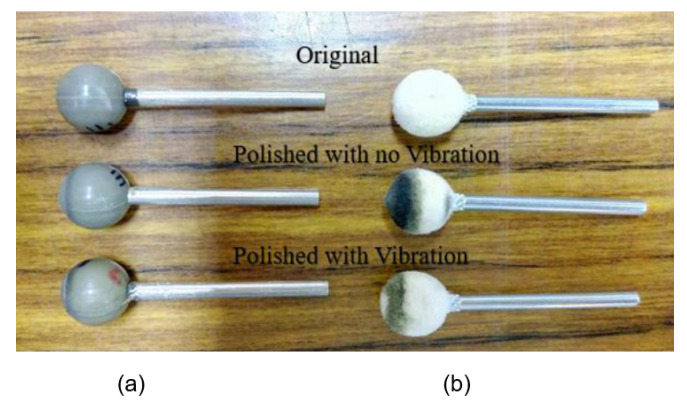
Comparison of the volumetric wear of the used polishing balls with vibration and with no vibration: (**a**) lab-made rubber polishing balls, (**b**) conventional wool polishing balls.

Tool wear reduction is one of the merits of the ultrasonic vibration-assisted machining as mentioned in the introduction. The volumetric wear investigation between the ultrasonic vibration-assisted polishing and the no-vibration-assisted polishing of the lab-made polishing balls from Type A to Type E and the conventional wool polishing balls has been carried out in this study. The solid models of the polishing balls used were constructed based on the 3D profile data of the polishing ball with wear, measured by a coordinate measuring machine. Creo Parametric 3D Parametric software (CREO 2016) has been used to construct the 3D solid models. The constructed solid models of the wool ball and the lab-made polishing balls from Type A to E, are shown in Figure 7. The volumetric wear of the ultrasonic vibration-assisted lab-made polishing balls ranges from 0.49% (Type A) to 0.73% (Type E), whereas the volumetric wear of the non-vibration-assisted lab-made polishing balls ranges from 0.56% (Type A) to 2.0% (Type E), as shown in Table 9. The improvement on the volumetric wear of the ultrasonic vibration assisted polishing of the lab-made polishing balls ranges from 12.64% (Type A) to 65.48% (Type E). The volumetric wear is increasing with the increase of the grain size and the concentration of the abrasives. The possible reason for that is the surface total bounding area decreases with the increase in the grain size and the concentration of the abrasives. The volumetric wear of the lab-made polishing balls is less than that of the conventional wool balls for both the ultrasonic vibration-assisted polishing and the non-vibration-assisted polishing. Figure 8 shows the comparison of the volumetric wear between the lab-made rubber polishing balls and the conventional wool polishing balls with vibration and with no vibration.

### 3.5. Application to the Surface Finishing of a Test Carrier with Saddle Surface

**Figure 9:**
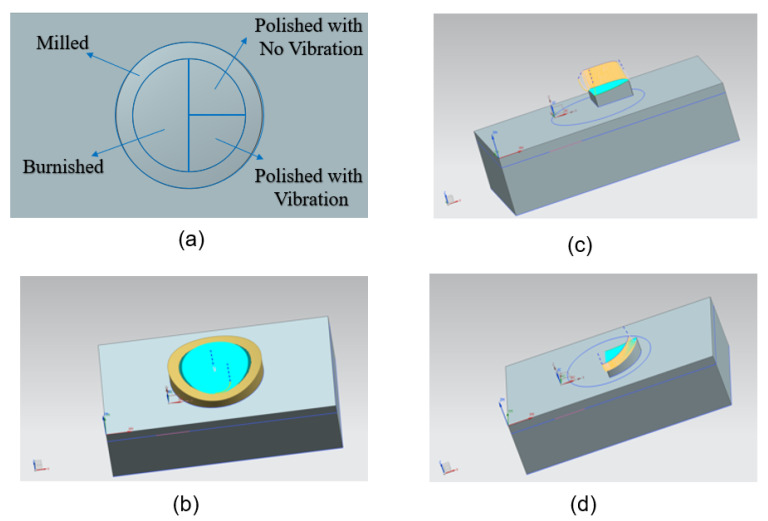
Constructed CAD model and machining path simulation of the test carrier with a saddle surface: (**a**) configuration of different areas, (**b**) fine-milling and burnishing, (**c**) polishing with no ultrasonic vibration, (**d**) polishing with ultrasonic vibration.

The determined suitable ball burnishing and ultrasonic vibration assisted ball polishing parameters for plane surfaces were sequentially applied to the surface finishing of a test carrier with a saddle surface. The Unigraphics NX10 CAD/CAM software (NX10) was used to construct the CAD model of the test carrier, as shown in Figure 9. Four areas on the CAD, namely fine milling, ball burnishing, vibration-assisted polishing, and non-vibration-assisted polishing, have been identified in Figure 9a. The machining path simulations for fine milling and burnishing, polishing with no ultrasonic vibration, and polishing with ultrasonic vibration, are implemented in Figure 9b–d, respectively.

**Figure 10:**
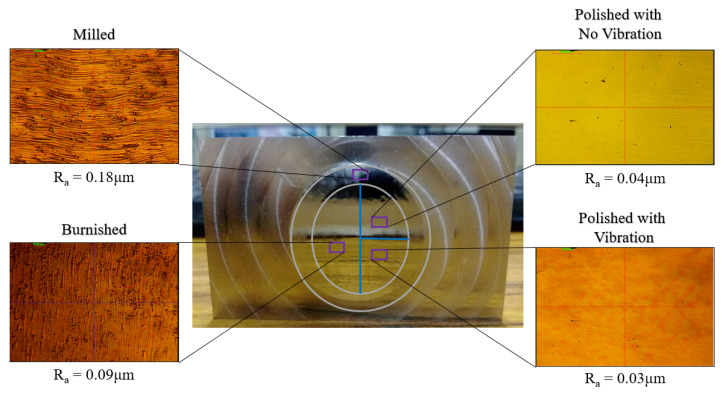
Measured surface roughness and surface texture (observed with a 30× optical microscope) of the fine-milled, burnished, polished with no ultrasonic vibration and with ultrasonic vibration on a carrier with saddle surface.

The ball burnishing process has been carried out after the fine-milling process. The ultrasonic vibration assisted polishing using the lab-made polishing balls have been sequentially applied to the burnished surface of the test carrier. The surface roughness R_a_ of the surface region on the annealed STAVAX test carrier (HRC = 22) was improved sequentially from about 0.18 μm to 0.03 μm, as shown in Figure 10. The surface textures on the fine-milled, burnished, and polished area have been observed with a 30× optical microscope. The surface roughness value Ra on the burnished surface was 0.09 μm on average. The surface roughness value Ra on the ultrasonic vibration-assisted ball polished surface was 0.03 μm on average, whereas that on the no-vibration-assisted ball polished surface was 0.04 μm on average. The surface roughness improvement of the 3D test object on the burnished surface was about 50%, and that on the vibration-assisted ball polished surface using the suggested E-C-B-A passes of the lab-made polishing balls was about 83% compared with the fine-milled surface.

## 4. Discussion

Although the used ultrasonic tool and the new lab-made polishing balls have been presented, there are still some issues to be discussed and investigated in the future, listed as follows:

1. Based on the experimental results of the multiple passes of ball polishing on the surface roughness improvement, in general, the more the passes used the better the improvement on the surface roughness. However, the results of the type E-C-B and type E-C-B-A showed a very small difference (0.016 μm) on the surface roughness. This implied that the variation in the same concentration of the abrasive with different grain size had no obvious influence on the surface roughness improvement. To reduce the polishing time, the surface roughness of Ra 0.04 μm on average was achieved by utilizing a number of Type E-C-B passes.
2. The volumetric wear of the lab-made polishing balls can be reduced by about 13% to 65% using ultrasonic vibration-assisted polishing. The mechanism for the reduction in the volumetric wear might be that the total sliding path had been reduced due to the intermittent contact between the vibrating polishing ball and the surface of the workpiece.
3. A constant force polishing process is suggested to be implemented regarding the wear of the polishing ball.
4. The diameter of the lab-made polishing balls was 12 mm. Considering the efficiency of polishing, different diameters of the polishing balls could be designed and fabricated to adapt the curvature of the workpiece. The cylindrical polishing pads with different diameters can also be utilized for a plane surface or a smoothed curved surface with small curvature in the future.

## 5. Conclusions

The ultrasonic-assisted surface finishing process of STAVAX mold steel on a 5-axis CNC machining center has been developed in this study by using new lab-made rubber polishing balls containing the aluminum oxide abrasive instead of traditional wool ball polishing equipped with a slurry container. The main results of this study can be summarized as follows:

1. Five types of NBR-based blanks for the polishing balls, Type A to Type E, have been made after NBR, aluminum oxide (Al_2_O_3_) abrasive, and some additives have been homogenously mixed by a blending machine. Five kinds of polishing balls with the diameter of 12 mm have been fabricated by the thermal forming processes using the lab-made molds, and some mechanical properties have been tested.
2. The effects of multiple polishing passes on the surface roughness improvement for the lab-made polishing balls has been investigated in this study. The multiple polishing process E-C-B-A resulted in the best outcome with a surface roughness of Ra 0.027 um on average.
3. According to the experimental results, the suitable combination of the ultrasonic vibration-assisted polishing parameters using the lab-made polishing balls was as follows: amplitude of 10 μm, frequency of 23 kHz, spindle speed of 5000 rpm, feed rate of 60 mm/min, stepover of 20 μm, depth of penetration of 180 μm, and polishing pass of E-C-B-A.
4. The volumetric wear of the Lab-made polishing balls is less than that of conventional wool polishing balls. The improvement in the volumetric wear of the ultrasonic vibration-assisted polishing of the Lab-made polishing balls ranges from 12.64% (Type A) to 65.48% (Type E), based on the calculation of the constructed CAD models of the used polishing balls.
5. The proposed suitable ball polishing parameters for a plane surface have been applied to the surface finishing of a test carrier with a saddle surface. The surface roughness improvement in the 3D test object on the burnished surface was about 50%, and that of the vibration-assisted ball-polished surface using the suggested E-C-B-A passes of the lab-made polishing balls was about 83%, compared with the fine-milled surface.

## Figures and Tables

**Table 1:** Chemical composition of STAVAX stainless mold steel (%) [31].

Composition	C	Si	Mn	Cr	V
%	0.38	0.9	0.5	13.6	0.3

**Table 2:** Specification of the ultrasonic tool [30].

Model	Specification
Holder Type	BT40
Holder Weight	3.5 KG
Input Power	110 V 220 V AC 50/60 H
Maximum Output	300W
Tool Speed	Max 6000 RPM
Tool Interface	ER-11
Ultrasonic Power	100~220 V
Operation Frequency	18~27 KHz
Vibration Average	<20 μm

**Table 3:** Concentration and abrasive size of aluminum oxide (Al_2_O_3_).

Type	A	B	C	D	E
Abrasive size (μm)	0.05 μm	0.3 μm	1 μm	3 μm	3 μm
Concentration (%)	20%	20%	30%	20%	40%

**Table 4:** Measure hardness, yielding strength, and static coefficient of the lab-made NBR-based blanks for the polishing balls.

Type	A	B	C	D	E
Hardness (HAS, Shore)	65 HAS	63 HAS	66 HAS	64 HAS	66 HAS
Yielding strength (N/mm^2^)	9.516 N/mm^2^	8.07 N/mm^2^	9.68 N/mm^2^	6.84 N/mm^2^	10.67 N/mm^2^
Static coefficient of friction	0.98	0.96	0.74	0.61	0.6

**Table 5:** Factors and levels of Taguchi’s experiments [30].

Factor	Level 1	Level 2	Level 3
A. Amplitude (μm)	6	10	---
B. Frequency (KHz)	18	20	23
C. Spindle Speed (rpm)	1000	3000	5000
D. Abrasive Diameter (μm)	0.05	0.3	1
E. Feed Rate (mm/min)	20	40	60
F. Stepover (μm)	20	40	60
G. Depth of Penetration (μm)	60	120	180
H. Abrasive Concentration	1:10	1:20	1:30

**Table 6:** Combination of the suitable ultrasonic vibration-assisted ball polishing parameters [30].

Factor	Level
A. Amplitude (μm)	10
B. Frequency (KHz)	23
C. Spindle Speed (rpm)	5000
D. Abrasive Diameter (μm)	0.3
E. Feed Rate (mm/min)	60
F. Stepover (μm)	20
G. Depth of Penetration (μm)	180
H. Abrasive Concentration	1:10

**Table 7:** Comparison of different number of the passes of the lab-made polishing balls on the surface roughness improvement.

No. of Pass	Test 1 (μm)	Test 2 (μm)	Test 3 (μm)	Mean (μm)
Burnished	0.13	0.13	0.13	0.130
E	0.08	0.08	0.08	0.080
D	0.10	0.90	0.90	0.093
E-A	0.08	0.07	0.07	0.073
E-C	0.07	0.07	0.06	0.067
E-C-B	0.04	0.05	0.04	0.043
E-C-B-A	0.02	0.03	0.03	0.027

**Table 8:** Combination of the ultrasonic-assisted process parameters for the lab-made polishing balls.

Factor	Level
A. Amplitude (μm)	10
B. Frequency (KHz)	23
C. Spindle Speed (rpm)	5000
D. Feed Rate (mm/min)	60
E. Stepover (μm)	20
F. Depth of Penetration (μm)	180
G. No. of Passes	E-C-B-A

**Table 9:** Volumetric wear of different polishing balls (original dia.: 838.6 mm^3^).

Volumetric Wear	Wool Ball	A: 0.05 μm (20%)	B: 0.3 μm (20%)	C: 1 μm (30%)	D: 3 μm (20%)	E: 3 μm (40%)
Volumetric wear with no vibration (mm^3^) (%)	33.5847 4%	4.7157 0.56%	7.9067 0.94%	12.5347 1.5%	11.1427 0.6%	17.197 2%
Volumetric wear with vibration (mm^3^) (%)	13.9207 1.66%	4.1557 0.49%	5.8127 0.69%	5.6557 0.67%	4.7537 0.57%	6.0907 0.73%
Improvement (%)	58.55%	12.64%	26.48%	60.07%	57.33%	64.58%

## Data Availability

Not applicable.
